# Factors influencing the integration of comprehensive sexuality education into educational systems in low- and middle-income countries: a systematic review

**DOI:** 10.1186/s12978-022-01504-9

**Published:** 2022-09-29

**Authors:** Malizgani Paul Chavula, Joseph Mumba Zulu, Anna-Karin Hurtig

**Affiliations:** 1 Department of Epidemiology and Global Health, Umeå University, 901 87 Umeå, Sweden; 2 School of Public Health, Department of Health Promotion and Policy Management, University of Zambia, Ridgeway Campus, P. O. Box 50110, Lusaka, Zambia

**Keywords:** Comprehensive sexuality education, Sexual reproductive, Health, And rights, Factors, Integration, Collaboration

## Abstract

**Background:**

Comprehensive sexuality education (CSE) plays a critical role in promoting youth and adolescent's sexual and reproductive health and wellbeing. However, little is known about the enablers and barriers affecting the integration of CSE into educational programmes. The aim of this review is to explore positive and negative factors influencing the integration of CSE into national curricula and educational systems in low- and middle-income countries.

**Methods:**

We conducted a systematic literature review (January 2010 to August 2022). The results accord with the Preferred Reporting Items for Systematic Reviews and Meta-analysis standards for systematic reviews. Data were retrieved from the PubMed, Cochrane, Google Scholar, and Web of Hinari databases. The search yielded 442 publications, of which 34 met the inclusion criteria for full-text screening. The review is guided by an established conceptual framework that incorporates the integration of health innovations into health systems. Data were analysed using a thematic synthesis approach.

**Results:**

The magnitude of the problem is evidenced by sexual and reproductive health challenges such as high teenage pregnancies, early marriages, and sexually transmitted infections. Awareness of these challenges can facilitate the development of interventions and the implementation and integration of CSE. Reported aspects of the interventions include core CSE content, delivery methods, training materials and resources, and various teacher-training factors. Reasons for adoption include perceived benefits of CSE, experiences and characteristics of both teachers and learners, and religious, social and cultural factors. Broad system characteristics include strengthening links between schools and health facilities, school and community-based collaboration, coordination of CSE implementation, and the monitoring and evaluation of CSE. Ultimately, the availability of resources, national policies and laws, international agendas, and political commitment will impact upon the extent and level of integration.

**Conclusion:**

Social, economic, cultural, political, legal, and financial contextual factors influence the implementation and integration of CSE into national curricula and educational systems. Stakeholder collaboration and involvement in the design and appropriateness of interventions is critical.

## Plain language summary

Adolescents and young people, especially those from low and middle-income countries (LMICs) face sexual and reproductive health (SRH) challenges. This review identifies factors that impact upon the integration of comprehensive sexuality education (CSE) into national curricula and educational systems in LMICs. A total of 442 original articles, published between 2010 and August 2022, were sourced from various databases. Thirty-four articles met the inclusion criteria. Social, economic, cultural, political, legal and financial factors are major enablers and barriers affecting the integration of CSE into educational systems in LMICs. Authorities in many countries are motivated to develop CSE interventions to help reduce SRH issues such as teenage pregnancies, early marriages, HIV/AIDS, and sexually transmitted infections (Yakubu and Salisu in Reprod Health 15:15, 2018;Herat in Reprod Health 15:1–4, 2018;). The rollout of CSE interventions is highly dependent on the availability of training resources, manuals, skilled teachers, and financing. In addition, the perception of teachers, students, politicians, and the community, influences the integration of interventions. This review shows that the involvement of key players in teaching is vital for successful implementation, as is building working relationships between teachers and community actors. Such collaborations enable the integrated delivery of SRH services. Insufficient monitoring and evaluation systems affects the quality of the implementation and therefore the success of CSE. We believe that community involvement in the development, implementation, monitoring, and evaluation of CSE is a major enabler for successful implementation and integration. Recognising and acknowledging the importance of political, social, legal and financial factors is critical for sustainable CSE integration.

## Introduction

Many low- and middle-income countries (LMICs) are integrating comprehensive sexuality education (CSE) into educational systems to promote youth and adolescents sexual and reproductive health and rights (ASRHR) as part of attempts to accelerate the attainment of universal health coverage and sustainable development goals by 2030 [1–4]. Meeting ASRHR is crucial for creating healthy socio-economic environments and promoting wellbeing in adolescents and young adults [4]. Yet achieving ASRHR presents major global challenges.

Adolescent girls and young women are highly susceptible to sexually transmitted infections (STIs) including human immunodeficiency virus (HIV) [4]. Many LMICs are facing several sexual reproductive health (SRH) challenges. World-wide one-tenth of all births are to females under the age of 20, and more than 90% of these occur in LMICs [5]. According to the United Nations Children’s Fund [2021], globally about 15 million girls are married before the age of 18 years. Evidence from LMICs shows that nearly one in three girls marry before the age of 18, and one in seven before the age of 15 [6, 7]. There are about ten million child marriages every year [6, 7]. This burden creates biological, emotional, social, and economic challenges. Risk factors associated with adolescent pregnancy include increased likelihood of low birth weights, preterm deliveries, infant respiratory diseases, and infant mortality [8]. Inadequate use or non-use of contraceptive services due to inaccessibility and avoidance of birth control methods, are major concerns. This occurs when women do not possess enough knowledge and confidence to make appropriate decisions on their SRH [11]. Moreover, the capacity to make informed choices is hindered by low socio-economic status, which in turn leads to a significant number of females selling sex or being exposed to sexual exploitation [9]. Drivers of unintended adolescent pregnancies include lack of education on SRH and more broadly limited CSE within national educational curricula. According to the United Nations Educational Scientific and Cultural Organization (2018), CSE is defined as:

“Comprehensive sexuality education refers to the curriculum-based process of teaching and learning about the cognitive, emotional, physical, and social aspects of sexuality. It aims to equip children and young people with knowledge, skills, attitudes, and values that will empower them to: realize their health, wellbeing, and dignity; develop respectful social and sexual relationships; consider how their choices affect their wellbeing and that of others; and, understand and ensure the protection of their rights throughout their lives.”

CSE has been previously described as an: “age-appropriate, culturally relevant approach to teaching about sexuality and relationships by providing scientifically accurate, realistic, non-judgmental information”[3].

Sustainable development goals and global health agendas recognise CSE as an important entry point for promoting adolescent health both as an end in itself, and as a means of improving the overall health and wellbeing of adolescents and young people [2, 3]. Many LMICs have adopted CSE into their formal education systems [10]. The curricula target children and adolescents enrolled in primary and secondary schools as a way of openly discussing adolescent health challenges [10]. There is extensive evidence that CSE creates opportunities for adolescents to acquire life skills and knowledge. Successful programmes have reduced gender inequality and gender-based violence (namely, intimate partner violence) [9]. CSE also provides opportunities for youth and adolescents to acquire the necessary information and skills on how their bodies function. It also demystifies sexuality and improves ability to make informed choices and decisions about SRH [11]. CSE can help reduce early pregnancies, unsafe abortions, and intimate partner violence, and promote increased condom use and self-efficacy. CSE can therefore improve SRH [11–13]. The programmes work best when they are socially and culturally sensitive [12, 14].

Yet full integration of CSE into the educational systems in LMICs remains a major challenge [15]. For instance, deep-seated discomfort about adolescent sexuality persists because of social, cultural, religious, structural, and institutional factors [16–19]. In many countries sexual abstinence is the dominant social message despite a body of evidence showing that abstinence education has limited or no effect on reducing risks [19–23]. Additionally, in some societies premarital sex is taboo. Trainee teachers not exposed to CSE in their own upbringing can lack the knowledge and skills to deliver CSE [24].

While there is extensive documentation on these challenges, little is known about the enablers and barriers affecting the integration of CSE into educational systems in LMICs. This review therefore explores factors influencing the implementation and integration of CSE into educational systems in LMICs.

## Conceptual framework

**Fig. 1:**
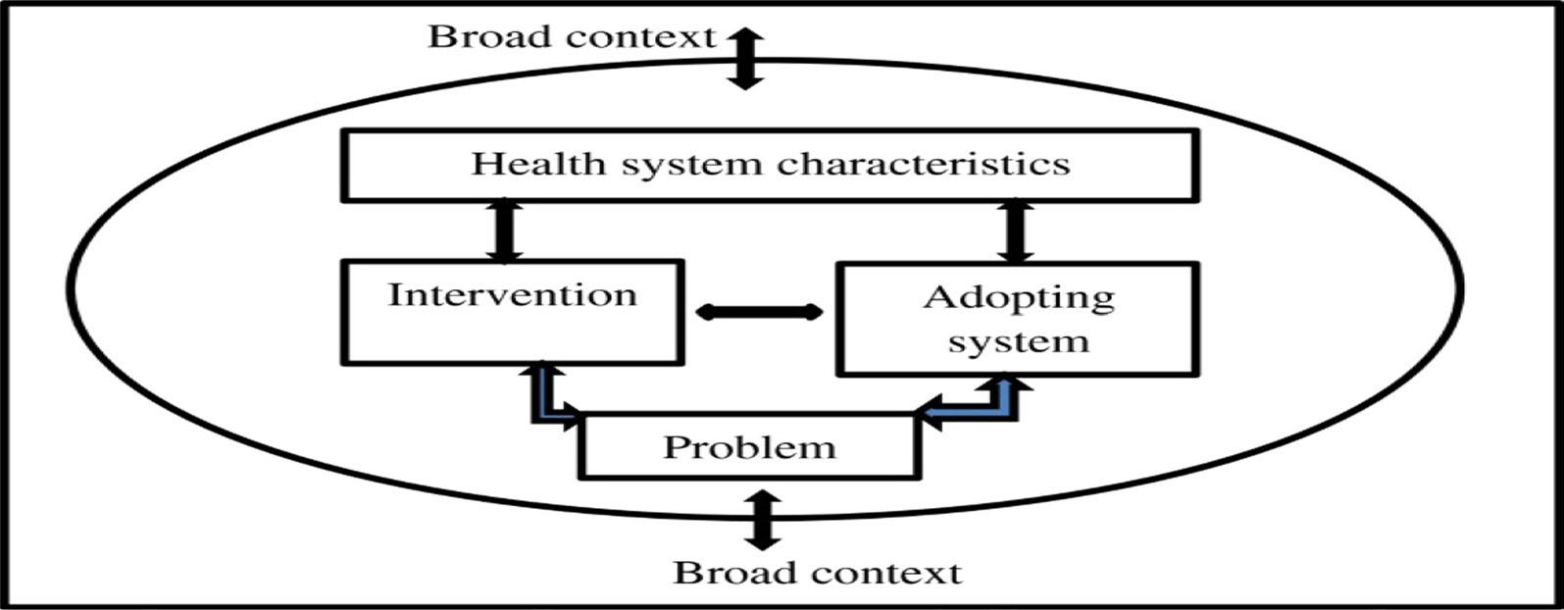
Conceptual framework for analysing the integration of CSE [25]

We adopted Atun et al.’s [25] conceptual framework because it provides a lens through which complex interventions such as CSE can be analysed. According to this framework, the integration and implementation of new health interventions are influenced by the nature of the problem being addressed, the intervention itself, the system of adoption, health system characteristics, and the broad context (Fig. 1). In the context of CSE, the *nature of the problem* includes discourse about impact and solutions to SRH challenges including high numbers of teenage pregnancies, early marriages, HIV/AIDS, STIs, and knowledge gaps. Attributes of the *intervention* include the core CSE content, methods of delivering CSE, training materials and resources and teacher training. Integration is influenced by the level of *adoption* and the program’s compatibility with *broad system characteristics*, such as links between schools and health facilities, school community-based collaborations, coordination of CSE implementation, monitoring and evaluation, as well as the *broad context* which includes, resources, policies and laws, international agenda and political commitment. A crucial overriding aspect is the way in which CSE is perceived by actors within the adopting system (e.g., teachers, learners, parents, religious and traditional leaders). This can either facilitate or inhibit the integration of CSE into education systems.

## Methods

### Study design/context

This review paper presents a systematic analysis of the factors relevant to the integration of CSE in the context of LMICs. We used the World Bank (2022) definition of LMICs as countries with a gross national income per capita of $US 1,085 or less in 2021. The review comprises 34 articles from 19 countries including Africa, Asia and Latin America. Twenty-four studies were conducted in eleven Sub-Saharan African countries: Kenya (n = 3), Tanzania (n = 4), Uganda (n = 4), Ghana (n = 3), Zambia (n = 3), Ethiopia (n = 2), Zimbabwe (n = 1), Nigeria (n = 1), South Africa (n = 1), Botswana (n = 1), and Mauritius (n = 1). The five studies from Asian countries were: Nepal (n = 1), Pakistan (n = 1), Philippines (n = 1), Myanmar (n = 1), Vietnam (n = 1). The remaining five were conducted in three Latin American countries: Peru (n = 2), Guatemala (n = 2), Nicaragua (n = 1). The review was structured in accordance with the Preferred Reporting Items for Systematic Reviews and Meta-Analyses guidelines known as PRISMA [26].

### Search strategy

We conducted an electronic search of the peer-reviewed literature in four databases: PubMed, Cochrane, Google Scholar, and Web of Hinari. We also located articles by searching reference lists in the included articles. We also conducted purposive searching of BMC and BMJ journals that focused on reproductive health, public health and sex education journals. The process was rigorous and iterative, and it continued until no additional studies were identified. Searches were conducted between August 2020 and August 2022. The key search terms included: barriers OR challenges AND enablers OR facilitators AND implementation OR integration AND “comprehensive sexuality education” OR “sexuality education” AND adolescents.

### Inclusion and exclusion criteria

The search was limited to English peer-reviewed publications for CSE programmes implemented in LMICs. Only literature published between January 2010 and August 2022 was included. During this period there was heightened international interest in the integration of CSE into school systems as a response to preventing SRHR challenges in many LMICs. Studies that documented the implementation of CSE in schools, process evaluation studies reporting lessons learnt that highlighted successes, and studies that discussed the challenges and opportunities of implementing CSE, were included. Studies were also included if they covered: stakeholders’ perspectives and experiences regarding implementation; the delivery of CSE by teachers; learners’ voices regarding the teaching of CSE, and parent and community perspectives. Studies that explored linkages and collaborations for CSE implementation and those that reported enablers and/or facilitators, such as teaching resources and funding opportunities, were also included.

### Study selection

**Fig. 2:**
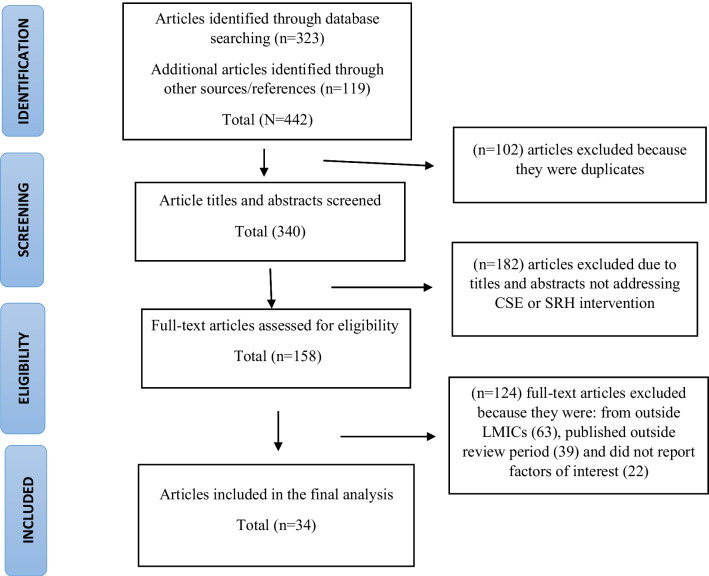
PRISMA diagram showing factors influencing the integration of comprehensive sexuality education into educational systems in low- and middle-income countries

The sample selection process is summarized in Fig. 2 [26]. The search first identified articles that broadly aligned with the concept of CSE. This resulted in 442 publications; 102 duplicate articles were excluded. We further screened the titles and abstracts of 340 remaining articles. One-hundred-and-eighty-two publications were excluded because they were unrelated to the subject (i.e., they did not address CSE or SRH interventions) leaving a total of 158. These articles were then considered for full-text reading. Of these, 124 were excluded because either they were not conducted in LMICs, they were published outside the review period, or they did not report upon the factors of interest. Finally, 34 articles met the inclusion criteria (Fig. 2). Quality assessment was performed using the critical appraisal skills programme [27]. A data extraction form was created in Microsoft Word Version 16 and used to extract information on key aspects (i.e., findings, designs, sample, data collection, analysis, and reporting).

### Data analysis and synthesis

Data from the selected publications were analysed using a thematic analysis approach [27] and NVivo software [QSR International UK, 20201]. This approach is consistent with the recommendation of the Cochrane Qualitative Review Methods Group [24]. A discussion between all the three reviewers/authors (MPC, JMZ and AKH) determined which data were to be extracted from the individual articles. The analysis process was guided by Atun’s conceptual framework. Firstly, codes were developed by the first two authors (MPC and JMZ) after reading and discussing an initial group of five articles. Secondly, the emerging coding framework was developed and discussed between all the three authors in order to identify similarities and differences between all studies and articulate the codes within logical meaningful themes. A coding framework was developed. This comprised broad themes based on the key components of the conceptual framework as in Fig. 1 [21]. We identified and explored themes and relationships within the coded data. We selected concepts, themes, and patterns by reading and re-reading the included studies. Data were imported and coded according to corresponding themes (Table 1) All authors reviewed the coding process on multiple occasions in order to reach consensus.

**Table 1:** Factors affecting the integration of comprehensive sexuality education

Themes	Sub-themes
Nature of the problem	•Higher prevalence SRHR problems
Attributes of the intervention	•Key SRHR issues that the CSE framework addresses •Methods of delivering CSE •Training materials and resources •Teacher training
System of adoption	•Observed benefits of CSE •Teachers’ characteristics and experiences •Learners’ experiences •Community perspectives •Parents' perspectives
Broad system characteristics	•Links between schools and community health systems •School and community-based collaboration •Coordination and implementation of CSE •Monitoring and evaluation of CSE
Broad context	•Commitment to an international agenda on CSE •Political commitment •Policies and laws •Resources

## Results

In this section, we present the factors shaping the integration of CSE into national curricula and educational systems in LMICs. The extent of the implementation and integration of CSE programs is analysed. This is followed by a presentation of the main results. This section is organised according to key elements in the conceptual framework i.e., characteristics of the problem, attributes of the intervention, the system of adoption, broad system characteristics, and broad context (Table 1).

### Study characteristics

Thirty-four studies were included for the final analysis. Of these, twenty-two were qualitative, four were programme evaluations, and five were mixed methods studies. The other three were document reviews. Detailed characteristics of the studies, their aims and major findings are given in Table 2.

### Level of integration of CSE programmes

This section presents a summary of selected cases of CSE programmes and reports on the degree and pattern of integration into national curricula and education systems. We found considerable variation across and within the programmes between countries. In some cases, the integration of CSE has progressed well, while in others it has been less than optimal [3, 10, 20, 24, 28, 29]. This limited integration was partly the result of the lack of training materials and opportunities, poor standardisation, and low teacher acceptance [30].

### Factors influencing the integration of CSE programmes into education systems

#### The nature of the problem

The nature of the problem influences the degree to which CSE interventions have been designed to address SRH-related gaps in the general education system. This section documents the major characteristics of the problem. Teenage pregnancies and marriages, STIs, and lack of knowledge of CSE were among the reasons for the interventions. Many authors of publications reported teenage pregnancies and early marriages as being major public health problems in LIMCs [5, 10, 31–33] and expressed concern about the unequal access to SRH knowledge amongst adolescents [10].

The literature indicates that lack of knowledge and negative environments regarding sexuality have made it difficult for adolescents and young people to make informed decisions. This is a major problem in LMICs [3, 31, 33, 34]. Unskilled teachers negatively impacted on the delivery of CSE and the response to SRH challenges [10, 33]. In addition, service provision*/*CSE interventions (e.g., counselling for adolescents) are insufficient [31]. Adolescents’ limited knowledge, misconceptions, and the non- or mis-use of contraceptives are factors associated with unintended pregnancy, access to modern contraceptives, unsafe abortions, violence (including gender-based violence), and puberty issues [32]. Adolescents and young people are facing high rates of STIs and HIV infections [15, 33]. Therefore, the development and implementation of CSE programmes is critical.

#### Attributes of the intervention

Because of increased awareness of the problems associated with SRH, many countries have introduced CSE. This section focuses on the pathway between attributes of the intervention (i.e., the CSE curriculum), its integration and implementation, and the perceived attributes of innovations such as CSE content, teacher training methods, and access to training materials.

##### Key SRHR issues that CSE framework addresses

Many LMICs have developed and designed CSE curricula to address the SRHR knowledge gap. CSE aims to increase adolescents’ and young peoples’ knowledge, develop positive attitudes and behaviour change, enhance skills and self-esteem, and empower young people to make informed choices concerning relationships, sexuality and their SRH [13, 30, 35]. Several studies report on CSE interventions that included contraceptive information (e.g., condom demonstrations and family planning) as strategies to prevent pregnancies and infections [28, 36–38]. Others describe content around reproduction, human development, puberty, hygiene management, and SRH [10, 32]. A rights-based approach promotes inclusiveness [10, 16, 30, 35]. Programmes have included discussions that aim to clarify values and attitudes towards culture and society [10]. A minority of the articles included information about how CSE has addressed the prevention of HIV/AIDS (Human Immunodeficiency Virus/Acquired Immune Deficiency Syndrome) [28, 36].

However, CSE interventions did not always cover topics such as personal and interpersonal development, sociability, relationships, and tolerance [10, 35], Few interventions dealt specifically with gender-based violence, access to legal systems, sexual harassment, adolescent pregnancies, and early marriage or abduction [3, 35]. This limits the environment in which students can challenge stereotypical gender boundaries. Content around sexual orientation (e.g., homosexuality, transgender non-discrimination and tolerance) was limited [28, 30].

Cultural, religious and societal factors curtailed the content of CSE in Islamic communities [3, 16, 30, 31, 35, 36] where abstinence-only education was taught [30]. Illustrations or images that were considered sexually explicit (such as reproductive organs) were dropped from the curricula [16, 36]. In some countries there were legal impediments for condom demonstrations and distribution [31]. Language was also one of the cultural barriers impeding CSE implementation. CSE materials were translated from English into local languages to improve comprehension and understanding. Yet terms such “self-esteem” and “decision making” for example, were not easily translated into local languages [3, 15, 30].

CSE was integrated into key subjects within the education system, such as physical, health education, basic science, home economics, religious education, biology, geography, and science [3, 10, 20, 24, 28, 29]. Some teachers taught CSE as part of their core duties. Yet CSE subjects were often elective and/or non-examinable and consequently were not regarded as important as compulsory examinable subjects. In other cases students who took particular elective subjects missed opportunities to take optional CSE lessons [10]. CSE was perceived as having the effect of diluting other subjects and diminishing the importance of the CSE curriculum [15]. If CSE was taught as a standalone subject this would have provided teachers with more time to explain SRH issues [15, 30, 33].

##### Methods of delivering CSE

Designing interactive approaches to the delivery of CSE can increase knowledge, develop positive attitudes, and enhance skills. Programmes have embraced a participatory learner-centred methodology [13]. These interactive methods included role-play, group discussions, drawing, songs, illustrations, brainstorming sessions, and short films [3, 23, 30, 37, 39]. These methods, which involved the depiction of real-life situations such as teenage pregnancies and complications during delivery, have engaged young people emotionally and promoted understanding [23, 39]. They create a safe space where sensitive topics can be discussed openly and comfortably [33]. Participatory methods have modified power relations between teachers and students, encouraged interaction, and helped involve learners [3]. Yet some teachers regard participatory and learner-centred approaches as difficult to implement [33]. Yet non-participatory techniques such as lectures or preaching (i.e., giving commands) to students [33, 37] reduce interaction and limit understanding.

##### Training materials and resources

The literature suggests that funding to support the development of teaching and learning material and resources has enabled the implementation of CSE in many LMICs [10, 15, 16, 29, 31, 33, 35, 36, 38, 40]. Financial support has been used to develop films, textbooks, curricula materials, teaching aids and images, training manuals, and other supplementary materials and resources [10, 16, 23, 29, 32, 35, 41]. Financial support has also enabled teachers to utilise the Internet and other Information Communication Technology services [23, 34]. Inadequate and inaccessible training materials, and the unavailability of library resources are cited as major barriers to CSE as is infrastructure (e.g., buildings housing classrooms) funding [10, 15, 16, 29, 33, 35, 36, 38, 40, 42]. Overcrowded classrooms impact on teachers’ ability to effectively deliver CSE lessons [29, 31].

##### Teacher training

Critical shortages of human resources were reported [31]. Some teachers had gaps in knowledge and skills leaving them unsuitable for teaching CSE [32, 41, 42]. CSE implementation required extensive teacher training in orientation, content-building, and pedagogical methodologies. Training has been rolled out at both pre-service and in-service levels [10, 13, 33] and decentralised from national to regional, district, and school levels [38]. This was intended to foster a greater sense of ownership and capacity amongst teachers and other stakeholders [36, 38]. It enables them to build capacity, and gain appropriate skills, time and commitment to ensure the successful scale-up of programmes [10, 35]. It also facilitates the creation of technical, managerial, and leadership teams for CSE implementation [35]. Numerous benefits of teacher training in CSE include recognition of the importance of CSE amongst educators and administrators. Many of the studies demonstrated that teacher training was one of the enabling factors in the integration of CSE into many schools [3, 10, 23, 30, 32, 33, 35, 37, 39, 40]. Moreover, trained CSE teachers were more motivated to teach CSE [40]. They better understood the dynamics of their class and were more likely to handle difficult relationships and cultural issues [32] compared with un- or under-trained teachers. Trained teachers also know how best to integrate CSE across different subjects [15].

The lack of teacher training was raised as a critical important issue [3, 10, 28, 33, 35, 40]. Untrained teachers were resistant to CSE because they had negative attitudes and experienced challenges in delivering CSE [28, 34]. Untrained teachers were rarely taught how to integrate CSE [15] and dropped topics resulting in a decline in CSE quality of CSE [28, 40, 41].

#### Adopting system

The perspectives and participation of actors such as teachers and school administrators, learners, health workers, and community members can influence the acceptability and implementation of national CSE programmes.

##### Observed benefits of CSE

Several benefits result from integrating and implementing CSE in schools [23, 34, 39]. They include imparting life skills including assertiveness, self-esteem, decision-making, critical thinking, and self-efficacy [23, 34, 39]. CSE enables adolescents to discuss sensitive topics such as menstruation openly with their peers and teachers [23]. There is evidence that students who had acquired CSE knowledge and skills were more likely to delay sexual initiation [31, 32] and use contraceptives [23, 31, 32]. There is also evidence that this has contributed to reduced numbers of teenage pregnancies, early marriages, STIs, and HIV [32]. CSE also helps discourage certain cultural practices and norms in communities where teenage pregnancies and early marriages have been traditional [23]. It enables young people and adolescents to grasp the importance of healthy relationships and helps them to appreciate the need to love and respect one another and protect themselves [34]. Evidence from studies in this review show that students who had never received any CSE were more likely to engage in high-risk sexual behaviours [32]. The following sections present the perspectives of different stakeholder groups regarding CSE.

##### Teachers’ characteristics and experiences

Teachers expressed a range of views regarding their experiences in implementing CSE. Some were highly motivated and appreciated the opportunity to teach adolescents and young people about the importance of the topic [13]. However, others held negative attitudes because of cultural and normative factors. Many teachers were more comfortable talking about abstinence, the promotion of morality, and life skills (e.g., communication, assertiveness, and decision-making skills) [10, 30, 38, 40]. In some cases sensitive topics (e.g., the mechanics and purpose of contraception, masturbation, and homosexuality) were skipped or dropped [3, 10, 30, 38]. This was partly because some teachers were not versed in the cultural backgrounds of their students, which meant that they held back from teaching CSE openly [33]. Moreover, individual teachers’ personal opinions and attitudes were not always compatible with some of the objectives of the CSE curriculum [13]. Teaching mixed-gender classes is not always acceptable [10]. There are views that teaching SRHR encourages premarital sex [37]. Communicating sensitive topics to young people not conversant in English was also difficult. Some teachers regarded the local terminology as insulting [10].

The gender of teachers also influenced the delivery of their lessons. For example, male teachers found it difficult teaching girls about some topics (e.g., menstruation and female reproductive and sexual organs) [31]. Hence girls and boys tended to be taught in same sex groups [31]. Girls were uncomfortable and shy when subjects like menstruation were raised [40]. Some teachers believed that CSE was not appropriate for girls in lower grades [10]. Many studies have shown that the teacher’s age can influence the willingness of adolescents and young people to accept CSE [30, 32, 33, 37, 39]. Older instructors were preferred because they were perceived to have had more experience in this area [39].

##### Learners’ experiences

CSE should go beyond the delivery of information to passive recipients (i.e., school students). Strategies that encourage active participation is integral to the empowerment of adolescents and helps them to make independent decisions with confidence [13]. Learning was more effective when interactive and participant-centred approaches (e.g., those incorporating audio-visual material) were used [23]. When discussing issues such as teenage pregnancies, complications during delivery and early marriages, students were emotionally engaged and learnt more easily [23, 39]. CSE helps adolescents to open up and share their personal experiences [43]. Participants reported that CSE changed their attitudes towards gender and sexuality and helped them to accept who they were [43]. Some said that CSE helped them to acquire assertiveness and self-esteem skills and have confidence when discussing sexuality [23, 43]. The acquisition of self-respect and life skills (including confidence) enables young people to make informed decisions about interpersonal relationships (including romantic relationships) and sexuality [43]. CSE also encourages young people to access SRH friendly services. Access to condoms is important for sexuality education and SRH promotion [44].

CSE increases knowledge and understanding of teenage pregnancies and early marriages. One important message was that contraception is seen as a means of preventing pregnancies and STIs [39]. Many adolescents said that they shared their CSE knowledge (e.g., of gender-based violence) with friends and family members. Some reported that they intervened in violent situations during or after the lessons [43].

##### Community perspectives

The literature includes discussion of community stakeholders’ positive and negative perspectives of CSE and how the participation of local people can facilitate integration into education systems. Some religious beliefs and values were reported as shaping the content used in teaching adolescents and young people [3, 10, 28, 31, 32, 34–37]. Religious beliefs supported communication between parents, teachers, and students on SRH-related issues [34]. Generally religious leaders were more open-minded and supported the teaching of CSE in schools [37]. However, Islamic, Hindu, and Christian (e.g., Catholics and Evangelicals) were resistant to the idea of students being taught topics such as kissing, masturbation, caressing, withdrawal, ejaculation, erection, and the use of contraceptive pills [3, 31, 34, 36, 37]. Aspects of Christianity (the Bible), Hinduism (Vedas), and Islam (the Qur’an) discourage sex before marriage and the discussion of sexuality amongst unmarried people [10, 23, 32, 34, 37, 40]. Contraception to prevent STIs and pregnancies was barred by some religions [3, 31, 37]; this was seen to encourage adolescents to engage in premarital sexual relationships (immoral behaviour) or “casual sex” and promote immorality and prostitution [3, 10, 36, 37]. Institutionalised religions have prevented teachers from adopting CSE [35–37]. Many political leaders, lawmakers and curriculum developers have opposed the implementation of CSE on the grounds that it corrupts young people and violates values by encouraging promiscuity, experimentation, and irresponsible sexual behaviour [45].

##### Parents' perspective

Parents are crucial stakeholders in shaping the implementation of CSE. Studies have reported that parents with strong cultural values and traditional beliefs have opposed the implementation of CSE in schools [10, 23, 30, 33, 36, 37, 40]. Parents have resisted the teaching of homosexuality, initiation ceremonies, menstruation, and contraceptive use on the grounds that they were socially and culturally inappropriate [10, 30, 33]. There are views that some topics were sacred and that only traditional counsellors (i.e., not teachers) were best suited to teach them [10, 23, 37]. Some opposed CSE because they believed it championed Western culture at the expense of local traditions [36]. Teachers succumbed to these views [37] in order to be seen to be supporting dominant cultural norms and values [33].

#### Broader system characteristics

Several studies have addressed the aspect of broader system characteristics, including links between schools and health facilities, collaboration, coordination, monitoring, and evaluation of CSE, and how these factors affected CSE implementation.

##### Links between schools and community health systems

The literature discusses facilitators or actors, outside the educational system, that have influenced the teaching of CSE in schools [31, 33, 35, 36, 39]. Some articles report teachers collaborating with community members, school associations, police and others outside the school system in order to publicly promote the importance of including critical content in sexuality education. The involvement of community health workers (CHWs), health care workers, police, and psychologists has strengthened the implementation of CSE [23, 31, 39]. Various actors such as the chiefs, political leaders and the media have appreciated the importance of teaching CSE in schools, and this has helped to break taboos associated with sexuality education [33, 37]. CHWs have assisted in changing cultural beliefs that oppose open discussion on sexuality education [33].

Some studies discuss the links between schools and health facilities [10, 29, 31]. Guidance and counselling coordinators have played critical roles in referring adolescents to health facilities where they could access SRH services [31]. Nurses and other healthcare workers provide screening for and treatment of STIs and comprehensive abortion services to adolescents [31]. Teachers have borrowed teaching aids from health facilities [10]. When issues were sensitive, they collaborated with CHWs and psychologists to help ensure that young people had access to accurate SRH information and services [31]. CSE programmes have created safe spaces such as youth-friendly services and community activities where students can talk about their experiences of sexual violence, both historical and ongoing [29, 30].

##### School and community-based collaboration

Teachers have collaborated with actors from the health sector, non-governmental organisations (NGOs) and communities in delivering CSE [30, 33, 36, 39]. In some studies, traditional, religious, and policy representatives met to make decisions regarding the implementation of CSE in schools [16, 35, 36]. Community health actors have held regular meetings to educate parents and members of the public who had opposed the teaching of CSE [31, 33, 34]. This has created a supportive environment and countered critical voices or negative social factors [16, 33]. In many cases collaboration with concerned parents and communities has enabled the contents of the curricula to be clarified before implementation [36].

The involvement of community leaders has deepened people’s understanding of CSE and broken the taboo of speaking about healthy sexuality [15, 16, 33, 34]. In many settings the mass media (e.g., the press, radio, and television) has helped to build positive public perceptions of CSE, discredit false statements or misconceptions [18], support teachers in teaching sensitive SRH content [31, 33, 35], and allay the idea that CSE was part of a foreign agenda [10]. Collaboration between actors has enhanced fidelity in delivering CSE content [31, 39]. Understanding the local context by engaging with stakeholders and creating adolescent-specific SRH curricula is crucial [16]. Some studies point out that sensitive topics were renamed after consultation. For example, the term sexuality education was changed to *life skills-based education* [16]. The inclusion of young people in curriculum development was also key to ensuring that content was tailored to their needs [10, 15, 28] because their views were not always taken into consideration [14]. Insufficient input from stakeholders including non-government organizations (NGOs) and youth organisations, has hindered the implementation of CSE [28].

##### Coordinating the implementation of comprehensive sexuality education

Existing coordinating mechanisms include national, provincial, district, and school-based steering committees and technical working groups [35]. The literature includes examples of how enhanced coordination improved planning, implementation, and monitoring in schools [15, 28, 30, 36, 38] and helped to oversee the integration of CSE into the educational curricula [35, 38]. In some cases technical working groups have been responsible for the routine management of CSE programmes [38]. National-level coordination involved regular channels of communication between the partners administering national implementation [28]. Multidisciplinary technical committees were composed of government, non-governmental, and international organisations [38]. Such coalitions have promoted universally accessible life skills-based education, or CSE [30, 35].

Nevertheless, structures for monitoring the implementation of CSE programmes were often absent [15, 28, 30, 36, 38]. A lack of coordination has meant that many partners (e.g., ministries of education, local authorities, and school administrators) worked in isolation [15, 28]. Consequently, parallel independent coordinating mechanisms (involving civil society and youth organisations and schools) were developed [15, 28]. Moreover, some stakeholders were unwilling to cooperate because they were competing for scarce resources [36]. Funders had different priorities; CSE delivery was dependent on ongoing funding [28]. This negatively affected the monitoring and coordination of CSE in some countries. Poor governance created confusion as to who was responsible for implementation [15]. Confusion has occurred regarding who was responsible for leading central governments, local governments and NGOs in implementing CSE [30]. This has arisen because of the lack of a formalised multi-sectoral approach to coordination [28, 36]. Studies report inconsistent and disorganised implementation, variability, and a lack of standardization. These factors negatively affect the quality and effectiveness of CSE implementation in schools [28].

##### Monitoring and evaluation of CSE

Tracking the progress of CSE through health information systems is crucial. Some countries created monitoring and evaluation frameworks to assess performance [15, 38]. Ministry of Education staff have conducted field visits during which they observed and monitored teachers’ delivery of CSE [15, 35]. However, monitoring and evaluation mechanisms are often weak or elementary [15]. There has been a lack clarity about who was supposed to collect data and when, and what tools were to be used [15]. Inadequate health information, and systemic issues have led to inconsistencies and discrepancies [15]. Schools in many countries did not report nationally on their CSE [15]. Monitoring of CSE was more complex in countries where inspectors were more interested in how it was integrated into subjects [15, 36].

#### Broad context

The literature covers how international and regional context has shaped the implementation of CSE locally, and, how international agendas and political, legal, and economic factors influence the process of integration in LMICs.

##### Commitment to an international agenda on CSE

Many countries that signed international protocols on SRH have made steps towards its implementation. There has been some commitment to an international agenda on SRHR. One example is the 1994 International Conference on Population and Development [15, 28, 30, 33, 35, 36, 38]. International commitments have been used by local stakeholders and civil society organisations to lobby and advocate for the introduction, implementation, and scaling-up of CSE and make governments accountable for ensuring that there are checks and balances regarding progress [28]. International protocols have created platforms where governments (through their ministries of education and health) have held regional meetings to promote collaboration and increase the number of government-run schools that were not delivering CSE by 2015 [28]. Ministries of education have been provided with technical and financial support from United Nations agencies, international NGOs, and experts outside government to develop fact-based policies and resist ideological negative pressures [28].

However, a lack of government commitment towards the proactive promotion of CSE and addressing opposing voices has impeded implementation, as have contradictions between health-related policies [28]. Donor funded CSE has been rejected by some actors (including teachers) who have side-lined implementation because they perceived it as a foreign agenda [10].

##### Political commitment

Implementation has also been affected by a lack of political will [28]. Very few countries were committed to SRHR or had national policies specifically dedicated to school-based CSE and its implementation [28]. However, some countries have institutionalised CSE for their schools [28, 29]. Yet few governments have shown openness to CSE, and this has slowed global progress [15, 28]. Some East African countries have banned the teaching of CSE [30]. The media has been responsible for some of the misrepresentation of CSE, and content did not comply with cultural and religious norms was censored [30].

##### Policies and laws

Some studies have reported that the policies and laws in some countries affected the implementation of CSE [28, 46], for example, by creating positive and enabling environments [28]. Some CSE scale-up strategies were formulated based on clear, innovative plans and actions [35]. Frameworks (with outlines of the topic) were established to guide CSE teaching and learning [10].

However, we observed that some policies and laws hindered CSE implementation [28, 46]. In certain countries, CSE information-sharing (regarding condom demonstrations and contraceptive use and SRH rights in particular) was prohibited by law [13]. There are also contradicting laws whereby some support and others prohibit the provision of ASRHR. This has negatively impacted upon CSE integration [47]. Discrepancies in policy direction between countries’ ministries of education and health suggest a lack of collaboration and coordination in the development of CSE policies and frameworks [28, 46]. For example, some ASRH policies in Ghana allowed young people aged 16 and over to access condoms [28] but the Ministry of Education prohibited the provision of SRH services in schools [28]. Health care workers and teachers have therefore experienced major challenges in attempting to provide CSE [46].

##### Resources

Some authors argue that the availability of resources has strongly influenced the implementation of CSE [5, 10, 15, 28, 31, 33, 36]. Insufficient teachers and other human resources has been an ongoing challenge. NGOs have provided experts to help governments manage interventions [10, 31, 36]. In some countries (e.g., Nigeria) CSE has been funded mainly through international organisations (e.g., the Global Fund) [36]. School managers at the local-level have played a critical role in mobilising teaching and learning resources [33]. However, funding has been disjointed, unpredictable [15], and sometimes withdrawn and this has impacted negatively on the continuity of implementation [28]. Some studies state that governments have not prioritised CSE funding [15, 28]. Insufficient resources and funding has prevented CSE workshops for open discussion of the issues [15]. School-based SRH programmes have been similarly negatively impacted [31, 36].

## Discussion

We used the conceptual framework developed by Atun et al. [25] to identify and explore factors influencing the integration of CSE into educational systems in LMICs. This review highlights the SRHR problem, the availability of training resources, stakeholder perceptions, and contextual issues. Our work shows the importance of reviewing and assessing a range of factors to better understand the complexity of CSE implementation in schools in LMICs.

Many LMICs have experienced SRHR challenges and there is recognition that CSE can help address these challenges. Stakeholders held varying views on the need to introduce CSE and the speed at which interventions should be rolled. Yet progress has been made in Ghana, Pakistan, Nigeria, Ethiopia and Kenya where there has been integration of CSE into national curricula and education system to address SRHR knowledge gaps [28, 31, 38, 40].

Studies on health innovations indicate that, in order for integration to be successful, the adopting system or context should be accommodating in terms of skills, resources, values, goals and regulations [25]. The training and the motivation of teachers delivering CSE are important factors for facilitating integration into school and community settings. In Nigeria, for instance, trained teachers helped to integrate CSE into the national curricula and the country’s education system [36]. In Ethiopia and Zambia, trained teachers have created a positive teaching and learning environment for their students [23, 30]. In Uganda and Ethiopia, teachers who adopted participatory methods (e.g., group discussion, drama, role play, and audio-visual resources) were similarly successful [23, 39, 40]. By contrast, the absence of trained teachers in Zimbabwean and Zambian schools meant that CSE was only partly integrated; many topics were either skipped or not included because they were deemed too controversial [10, 33]. The lack of available teaching, learning and financial resources has hindered the implementation and integration of CSE in Guatemala and many sub-Saharan African countries [15, 28, 34, 40].

**Fig. 3:**
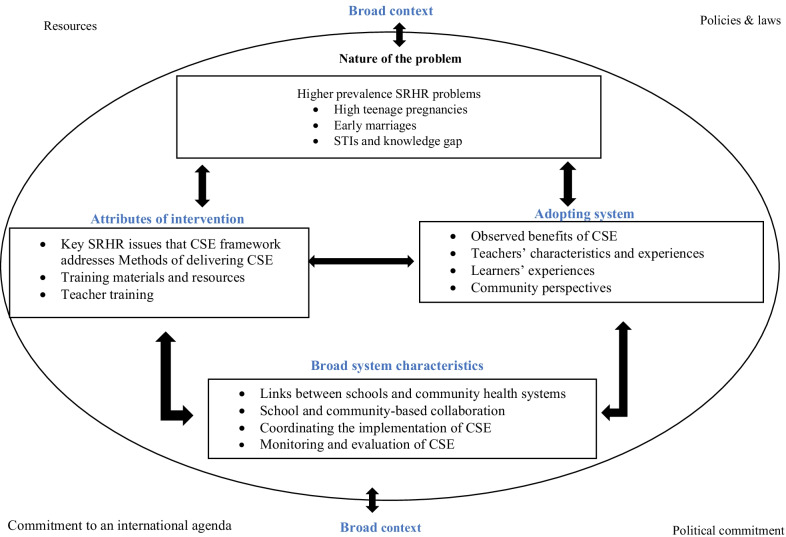
Factors influencing the integration of comprehensive sexuality education programmes into educational systems

This review illustrates how contextual factors and teachers’ gender can play a part in the implementation and integration of CSE. In Ethiopia, Ghana, Kenya, Peru and Guatemala, teachers and students of the same gender had a positive effect [15]. Studies conducted in Southern and West Africa (namely, Botswana, Zimbabwe, and Nigeria) concluded that teachers with negative attitudes and perceptions regarding CSE hindered progress [33, 36, 48]. Similarly, the reaction of community and religious leaders and parents had a significant impact on the acceptance of CSE. In Southern, West, and East Africa and Pakistan, local contextual factors, including religious and cultural values, limited the spread of CSE [10, 36, 39, 40]. Topics such as homosexuality, initiation ceremonies, and contraception conflicted with dominant religions and cultural practices [30, 48]. Collective action in the delivery of SRHR services in community health systems can promote ownership, trust and sustainability for the integration of CSE interventions [49–51]. It is important for interventions to suit the local context. This is shown in Fig. 3.

Broader contextual factors have affected the extent to which CSE was implemented and integrated. Politicians in Ghana, Peru, Kenya, and Guatemala were more accepting of CSE [28] than those in Zambia and Uganda [10, 30] and this influenced take-up. Socio-economic development and governmental commitment to CSE shaped understanding of the nature of the problem and the epidemiology of SRHR—as did interventions and support mechanisms (e.g., training materials distribution, capacity building in human resources, implementation financing, and monitoring and evaluation) in schools and community health systems. However, this review shows that government commitment to the global CSE agenda has led to collaboration and financial and social accountability. In many countries this has also shaped the level of implementation and integration. Moreover, these broader factors also influenced the integration of CSE in terms of adoption and implementation, collaboration and coordination, and monitoring and evaluation. Figure 3 shows the integration of CSE into the national curriculum and educational systems in LMICs based on the conceptual framework developed by Atun et al. [25].

The review also identifies some of the many opportunities for the involvement of various community actors in the integration of CSE at the micro-level. Engagement has helped to leverage existing structures, networks, and decision-making processes in the delivery of CSE in community settings and schools. These relationships were also influenced by broad system and contextual factors.

Collaboration (e.g., in the form of community engagement) was identified as a key factor influencing integration across systems. For example, teachers in Ethiopia networked with parent-teacher-student associations, local NGOs, and local government bodies, who supported and contributed to the optimal integration of CSE programmes [40]. This allowed the schools access to financial, material, and technical resources [36]. In Uganda, the community supported the full integration and implementation of CSE in its schools [52]. Collaboration between schools and community actors also created opportunities to educate the public about the importance of CSE. Local actors in Ghana, Botswana, Zambia, and Nigeria were less involved, so integration was only partially realised [10, 31, 48]. This weak collaboration made it difficult to establish links between SRHR and service delivery for adolescents and young people.

In Uganda, the proper coordination of school-based CSE activities facilitated links with community support systems for ASRH [30]. Effective coordination, monitoring, and evaluation has enabled administrators to comprehend the challenges associated with the implementation of CSE, including documenting limited coverage in secondary schools and nipping socio-cultural impediments in the bud [30, 36]. Some studies point out that schools with monitoring and evaluation mechanisms (in the form of tracking sheets) helped to provide feedback from students to teachers [30, 48]. The availability of local administrative support and supervision at the school level facilitated full integration in some countries. The lack of monitoring and evaluation systems was a hindrance in others [28]. In Ghana, Peru, Kenya, and Guatemala, for example, the absence of a multi-sectoral approach between education and health sectors resulted in erratic and disorganised implementation and undermined the quality and effectiveness of CSE [28]. In these countries monitoring and evaluation processes were partially integrated with staff from the regional ministries of health and education jointly who were undertaking these tasks.

## Limitations and strengths of the study

We acknowledge that there are limitations to this review. While we attempted to conduct a comprehensive search of the literature, we may have missed some relevant studies. We endeavoured to mitigate this possibility by conducting several searches between August 2020 and August 2022 as well as searching the references of the included publications. Another limitation was that all the studies examined were in English, this being the dominant language used for research publications. However, we attempted to maximise coverage by including papers reported within published systematic reviews, some of which were not retrieved from the initial search. The majority of the studies were conducted in countries in the African continent and thus caution should be exercised when generalising the results to other contexts. However, the issues raised were consistent across multiple countries and studies. This review does not document the role of collaboration in CSE integration. Further research is required to understand the nature and patterns of collaboration.

## Conclusion

Promoting ASRH through the integration of CSE into national curricula and educational systems remains a big challenge in many LMICs. The major drawback lies in contextual factors and inadequate stakeholder involvement. It is paramount that these limitations are addressed through inclusive collaboration in the planning, implementation, monitoring, and evaluation of CSE in educational systems. Collaboration is essential as it facilitates the co-identification of SRH problems and the co-design of CSE interventions and connectedness in the mobilisation of resources (e.g., training materials, human resources, and funding). Healthy partnerships are also essential for strengthening the links between SRH service delivery and existing structures and networks.

Collaboration also encourages stakeholder buy-in, for example by reducing resistance and helping to co-create CSE interventions that are compatible with the local and broader context. The engagement of different actors promotes ownership and enhances the ability of the community to provide social accountability mechanisms through checks and balances. This suggests the need for future studies that map key stakeholders in relation to their roles to facilitate documenting baseline contextual factors at the beginning of the integration process. It is crucial to have agreement on their expected roles for CSE integration into the education systems.

Policies and broad contexts play crucial roles in shaping the integration process. However, limited evidence exists on how SRH related policies influence the integration of CSE. Rigorous evidence-based methodologies are needed to guide the documentation of policies in relation to how they hinder or support CSE implementation in LMICs. Future studies should also focus on describing prevalence, explaining relationships, and the generalization of issues affecting the integration of CSE into different educational systems. Finally, there is a need for a comparative analysis of factors shaping the integration of CSE not only in LMICs, but also in high-income countries.

## Data Availability

The study data can be requested from the author. The articles for this review can be made available upon request.

## Appendix

See Table 2 Study characteristics

**Table 2:** A systematic review of factors influencing integration of comprehensive sexuality education in educational systems in low- and middle-income countries

No	1st Author/Year/[Citation]	Country	Study type/design	Study aim	Study participants	Key issues/findings
1	Mbeba et al. (2012)	Tanzania	Qualitative case study	This study aimed at gaining insights on barriers to the utilization of SRHS in Mtwara district	Girls (10–18 years), community leaders and adults	The study revealed that a good number of health facilities do not have skilled service providers (SPs) on sexual reproductive health rights-included; education, family planning and voluntary counselling and testing. However, the services were inaccessible due to lack of privacy, confidentiality, equipment’s and negative attitudes from SPs. Initiation ceremonies, early marriages and gender disparities were mentioned as social-cultural barriers to SRH rights
2	Venkatraman Chandra-Mouli, (2018) [16]	Pakistan	Programme evaluation	This review aims to answer the following questions: (1) How did Aahung and Rutgers Pakistan work to understand Pakistani society and culture and shape their programmes to build community support? (2) How did Aahung and Rutgers Pakistan overcome resistance to their efforts?	Documents and publications	The success of Aahung and Rutgers Pakistan was grounded in their readiness to understand the nuanced context within the communities, collaborate with groups of stakeholders—including parents, school officials, religious leaders, media personnel, and adolescents themselves—to ensure support, and stand up to forces of resistance to pursue their goals. Specific strategies included working With communities to select content, tactfully selecting and framing issues with careful consideration for sensitivities, engaging adolescents' influencers, strengthening media presence, showcasing school programmes to increase understanding and transparency, and choosing opportune times to introduce messages
3	Herat et al. (2018) [2, 18]	Uganda	Qualitative study (natural setting)	exploring the challenges faced by male and female persons with physical disabilities in accessing SRH services in Kampala, Uganda	Advocates for and persons with disabilities	The study findings show that PWPDs face a multitude of challenges in accessing SRH services including negative attitudes of service providers, long queues at health facilities, distant health facilities, and high costs of services involved, unfriendly physical structures and the perception from able-bodied people that PWPDs should be asexual
4	Keogh et al. (2018) [15]	Ghana, Kenya, Peru and Guatemala	Mixed Method study (natural setting)	This paper analyses the challenges to the implementation of national CSE curricula in four LMICs: Ghana, Kenya, Peru and Guatemala	It presents qualitative findings from in-depth interviews with central and local government officials, civil society representatives, and community level stakeholders ranging from religious leaders to youth representatives	Programme planning-related challenges included insufficient and piecemeal funding for CSE; lack of coordination of the various efforts by central and local government, NGOs and development partners; and inadequate systems for monitoring and evaluating teachers and students on CSE Curriculum implementation-related challenges included inadequate weight given to CSE when integrated into other subjects, insufficient adaptation of the curriculum to local contexts, and limited stakeholder participation in curriculum development
5	Browes (2015) [3]	Ethiopia	Qualitative study (natural setting)	Understanding CSE, culture and gender: the effect of the cultural setting on a sexuality education programme in Ethiopia	Teachers and students, both male and female	Results show that CSE teachers and students, both male and female, were able to discuss issues of sexuality after the course. However, the cultural context was seen to affect interpretation of programme information, influencing the nature of this discussion. For impactful implementation, it is recommended that sexuality education aims to engage with and involve the wider community, to reduce contradictory messages and increase programme support. Furthermore, teachers should undergo extensive and comprehensive pre-programme training that addresses their attitudes and values, not just their knowledge
6	Emambokus et al. (2019) [34]	Mauritius	Qualitative case study (natural setting)	The aim of the study was to explore parents’ and teachers’ perspectives of socio-cultural factors that can act as enabling factors or potential barriers	parents and teachers	Analysis of the interview transcripts revealed that the enabling factors were perceived as the importance of school-based SE by parents and teachers, contribution of external organizations, and a two-way communication process with adolescents. The potential barriers were perceived as a resistance from some teachers and students, the gender of the parent, and religion. Generation gaps and information communication technology were found to be both enablers and barriers. The findings have implications for the design and implementation of school-based SE within a multicultural context and pave the way for similar studies on a larger scale
7	Mhlauli and Muchado (2015) [48]	Botswana	Qualitative study using grounded theory (natural setting)	The purpose of this study was to explore the teachers’ and students’ perceptions on issues of sexuality in primary schools in Botswana	teachers and students	The major finding of the study revealed that there is an intergenerational conflict of ideas and views between teachers’ and students’ pertaining to issues of sexuality in primary schools in Botswana
8	Chavula et al. (2021) [23]	Zambia	Qualitative study (CRCT)	Experiences of teachers and CHWs implementing sexuality and life skills education in youth clubs in Zambia	CHWs and teachers	The teachers and CHWs reported that the use of participatory approaches and collaboration between them in implementing CSE enabled them to increase girls' and boys' participation youth clubs. However, some teachers and CHWs experienced practical challenges with the manuals because some concepts were difficult to understand and translate into local language. The participants perceived that the youth club increased knowledge on CSE, assertiveness and self-esteem among the learners. Training and providing a detailed teaching manual with participatory approaches for delivering CSE, and collaborative teaching enabled teachers and CHWs to easily communicate sensitive SRH topics to the learners
9	Le Mat et al. (2019) [40]	Ethiopia	Qualitative study (natural setting)	We aim to improve understanding of the ways in which teachers enact and re-contextualise CSE policy, and their reasons for doing so	Young men and women, teachers, school administrators, parents/community and local leadership	Implementation of CSE was influenced by School context, Socio-economic environment, Relations with the community, Socio-cultural roles and responsibilities
10	Bylund et al. (2020) [46]	Tanzania	A qualitative and cross-sectional study (natural setting)	This study has aimed to explore and understand health professionals’ perceptions and attitudes regarding the provision of adolescent sexual and reproductive health care in a selected national sexual and reproductive health programme in the Arusha region and Kilimanjaro region, Tanzania	Health care workers (nurses, social worker, clinical officer, midwife, HIV officer etc.)	Concern about the stigma directed towards adolescents, Over-medicalisation of Services, Difficulty involving adolescent Males, Ambiguous policies and contradictory messages
11	Muhwezi et al. (2015) [52]	Uganda	Qualitative study (natural setting)	Exploring perceptions and experiences of adolescents, parents and school administrators regarding adolescent-parent communication on sexual and reproductive health issues in urban and rural Uganda	Students, school administrators and teachers	Parents to be foundational for a healthy adolescent- parent communication, onset of menstruation and perceived abortion in the neighbourhood, Peers at school and mass media were perceived to the main source of sexuality information
12	Chandra-Mouli (2013)	Tanzania	Document review	Exploration of Standardizing and scaling up quality adolescent friendly health services in Tanzania	Review of plans and reports from the MOHSW	There was no standardized definition of CSE, poor quality of the CSE being provided by some organizations, problems by mapping existing CSE services, to improve their quality and expand their coverage, integrating CSE within wider policy and strategy documents and programmatic measurement instruments
13	De Haas and Hutter (2019) [37]	Uganda	Qualitative Study (natural setting)	Teachers’ conflicting cultural schemas of teaching comprehensive school-based sexuality education in Kampala, Uganda	Teachers and NGOs staff, religious leaders, public staff, students and private organisations	Young people are both innocent and sexually active; sexuality education both encourages and prevents sexual activity; and teachers need to teach sexuality education, but it is considered immoral for them to do so. In countries such as Uganda, supportive school regulations and a mandate from society could help teachers feel more comfortable adopting comprehensive approaches to sexuality education
14	Panchaud et al. (2018) [28]	Ghana, Peru, Kenya and Guatemala	Qualitative case study	Exploration towards CSE: a comparative analysis of the policy environment surrounding school-based sexuality education in Ghana, Peru, Kenya and Guatemala		The study shows that all four countries benefit from a policy environment that, if properly leveraged, could lead to a stronger implementation of CSE in schools However, each faces several key challenges that must be addressed to ensure the health and wellbeing of their young people. Latin American and African countries show notable differences in the development and evolution of their CSE policy environments, providing valuable insights for programme development and implementation
15	Kunnuji et al. (2020) [36]	Nigeria	Qualitative study	Understand the Variable Implementation of Sexuality Education in Three Nigerian States	government officials, Non-governmental organizations, Funders, Researchers, Principals/Teachers and civil society opponents of sexuality education	In summary, the interaction of socio-cultural context, domestic champions, adaptive capacity of state bureaucracies, and international funders explains variable implementation of family life health education FLHE. The Nigerian experience highlights the need for sexuality education proponents to anticipate and prepare for local opposition and bureaucratic barriers. Socio-cultural/ religious [Islam] context was a strong opposition to the implementation CSE. Cosmopolitan socio-cultural context in Lagos presented a supportive atmosphere for the implementation of the curriculum
16	Zulu et al. (2019) [10]	Zambia	Qualitative case study (natural setting)	This paper explores how teachers perceive the curriculum and practice discretion when implementing the CSE in mid-level schools in Nyimba district in Zambia	teachers	This discretion implies holding back information from the learners, teaching abstinence as the only way of preventing pregnancy or cancelling sexuality education sessions altogether. Teachers’ choices about the CSE programme were linked to lack of guidance on teaching of the curriculum, especially with regards to how to integrate sexuality education into existing subjects. Limited prioritization of CSE in the educational sector was observed. The incompatibility of CSE with local norms and understandings about adolescent sexuality combined with teacher-parent role dilemmas emerged as problematic in implementing the policy. Limited ownership of the new curriculum further undermined teachers’ motivation to actively include CSE in daily teaching activities. Use of discretion has resulted in arbitrary teaching thus affecting the acquisition of comprehensive sexual and reproductive health knowledge among learners
17	Gudyanga et al. (2019) [33]	Zimbabwe	Qualitative study (natural setting)	Exploring Zimbabwean secondary school Guidance and Counselling teachers teaching sexuality education in the HIV and AIDS education curriculum	Guidance and Counseling teachers	Engaging with culture in relation to healthy sexuality, Strengthening the guidance and counselling teachers Professionally, Leadership support, Creating a space for teacher reflexivity and agency to teach sexuality education, sensitised towards cultural diversity, to teach and not to preach, Seeing possibilities for a new participatory method of teaching
18	Godia et al. (2013)	Kenya	Qualitative study (natural setting)	health service providers’ experiences in Sexual reproductive health service provision to young people in Kenya	Clinical officers Nurse/Midwives Counsellors	The majority of health service providers were aware of the youth friendly service concept but not of the supporting national policies and guidelines. HSP felt they lacked competency in providing SRH services to young people especially regarding counselling and interpersonal communication. HSPs were conservative with regards to providing SRH services to young people particularly contraception. HSP reported being torn between personal feelings, cultural and religious values and beliefs and their wish to respect young people’s rights to accessing and obtaining SRH services
19	Tabong et al. (2018) [31]	Ghana	Qualitative Study (natural setting)	Acceptability and stakeholders’ perspectives on feasibility of using trained psychologists and health workers to deliver school-based sexual and reproductive health services to adolescents in urban Accra, Ghana	adolescents aged 12–17 years, teacher group, teachers, managers of schools, health workers, clinical psychologists, as well as ASRH programme managers	Acceptability of school-based SRH services, challenging for parents and/or teachers to provide adolescents with SRH information, have health workers and psychologists provide SRH information and services to adolescents in school, many of the respondents disagreed with distribution of condoms in schools as they believed that availing condoms would encourage adolescents to experiment with sex
20	Kemigisha et al. (2019) [30]	Uganda	Qualitative study (programme evaluation)	Process evaluation of a CSE intervention in primary schools in South Western Uganda	students, teachers, student educators and parents	Delivery of the anticipated 11 CSE lessons occurred in all target schools with moderate to high student attendance, however the duration of sessions was often shorter than planned. Facilitating factors for implementation included establishment of a community advisory board, use of multiple interactive delivery methods and high acceptance of the programme by key stakeholders. Socio-cultural norms, geographical access, time constraints and school related factors were barriers
21	Renju et al. (2010) [29]	Tanzania	Qualitative study (programme evaluation)	Scaling up a school-based sexual and reproductive health intervention in rural Tanzania: a process evaluation describing the implementation realities for the teachers	teachers, head teachers, ward education coordinators and school committees	Training was well implemented and led to some key improvements in teachers’ ASRH knowledge, attitudes and perceived self-efficacy, with substantial improvements in knowledge about reproductive biology and attitudes towards confidentiality. The trained teachers were more likely to consider ASRH a priority in schools and less likely to link teaching ASRH to the early initiation of sex than non-trained teachers. Facilitating factors included teacher enjoyment, their recognition of training benefits, the participatory teaching techniques, support from local government as well as the structured nature of the intervention. Challenges included differential participation by male and female teachers, limited availability of materials and high turnover of trained teachers
22	Rijsdijk et al. (2014)	Uganda	mixed methods design (programme evaluation)	Implementation of The World Starts with Me, a comprehensive rights-based sex education programme in Uganda. Health education research	Teachers,	Supportive factors of implementation (their students, head teachers, school management, and other teachers. Teachers felt less supported by parents, health-care providers and religious institutions and supported by their own family). (Non) supportive beliefs and norms towards rights-based sex education [disapproved of their students having sexual intercourse and were in favour of abstinence until the age of 18, preferably until marriage]. Some teachers said they did not promote condom use because of their religious views
23	Chirwa-Kambole et al. (2020) [39]	Zambia	Qualitative case study (CRCT)	Acceptability of youth clubs focusing on comprehensive sexual and reproductive health education in rural Zambian schools: a case of Central Province	Students and teachers	The perceived advantage and simplicity of the clubs related to the use of participatory learning methods, films and role plays to communicate sensitive reproductive health information made the learners like the youth clubs. Further, the perceived compatibility of the content of the sessions with the science curriculum increased the learners’ interest in the youth clubs as the meetings also helped them to prepare for the school examinations. However, cultural and religious beliefs among teachers and parents regarding the use of contraceptives complicated the delivery of reproductive health messages and the acceptability of youth clubs’ information among the learners
24	Adekola et al. (2021) [44]	South Africa	Qualitative study	Exploration Of Addressing Learner-Centred Barriers to Sexuality Education in Rural Areas of South Africa: Learners’ Perspectives on Promoting Sexual Health Outcomes	Learners (grade 10–11)	Learner-centred barriers to effective school-based sexuality education identified in this study were attitudes, age disparity, psychological status, peer pressure, socio-economic status, the exploratory attitude of learners, media, lack of role models, previous experiences, socio-economic status, and lack of parental love. These factors could reduce good sexual health. Learner-targeted interventions such as campaigns, using guest professionals, condom distribution, videos, on-site family planning, formal demonstrations, and on-site counselling could address these barriers
25	Ogolla et al. (2019) [41]	Kenya	Mixed method study	Assessment of the implementation of CSE in Kenya	Teachers	The study found low awareness in key topics such as HIV/STIs, condom use, benefits of abstinence and contraception. Most teachers were not trained in CSE, and CSE is not included in the curriculum. Personal biases, opinions and values related to sexuality education threaten the delivery of CSE. Resource materials are also unavailable. The study concluded that teachers acknowledged the need for CSE. However, its delivery is severely inhibited by lack of training, non-inclusion of CSE in the curriculum, inadequate time allocation for CSE lessons, and lack of teaching resources
26	Pokharel et al. (2021) [45]	Nepal	Document review	Understand Current Perspectives on Adolescent Sexuality Education in Nepal:	Documents	sex education in schools is a sensitive issue. Adolescents in Nepal are affected by taboos surrounding sexuality which are the main socio-cultural challenges to gain sexual health education. Lawmakers and curriculum developers claim that sexuality education corrupts young people and violates “Nepalese values”, leading to promiscuity, experimentation, and irresponsible sexual behaviour
27	Zaw et al. (2021) [19]	Myanmar	Mixed method	determine the preferences for type of sexuality education at high schools, compare the level of knowledge of reproductive health among actors	Students, parents and teachers	Focus group discussions were held to explore their insights on sexuality education for Reproductive health knowledge was low, particularly among students, and particularly with respect to knowledge of sexually transmitted infections. Cultural issues, training and manpower emerged as key themes from the focus groups. Over half of the students said that they were not receiving any form of sex education at school. Reproductive health knowledge was unsatisfactory among all participants reflecting the insufficiency of current sexuality education classes. Training and support for teachers should be provided
28	Melgar (2021) [47]	Philippines	Document review	Assessment of country policies affecting reproductive health for adolescents in the Philippines		Contradicting laws, some support and others prohibit provision of ASRHR services. Non-discrimination in information and services. Integration of contraceptives, including emergency contraception, into essential medicines. Teacher training in delivery of adolescent sexual reproductive health is still neglected
29	Ha et al. (2021) [42]	Vietnam	Qualitative study	The provision of sexual and reproductive health education to children in a remote mountainous commune in rural Vietnam	Parents, youth leaders and teachers	The head of the Youth Union and lecturers at secondary school stated that they were not capable of providing SRH training as none of their staff were specifically trained in SRH and they had no access to appropriate SRH educational materials. Meanwhile, there was no public library or bookstore in the commune where young people could have access to SRH reading material. There is a major gap between the SRH education needs of parents and children in remote rural areas of Vietnam and the resources required to address these needs
30	Larsson et al. (2021)	Nicaragua	Qualitative study	Exploring sexual awareness and Decision-making among adolescent girls and boys in rural Nicaragua	Girls	Girls turned to parents on topics of sexuality while boys turned to peers. Social stigma hinders adolescent’ access to sexual and reproductive health services. Social media appears to influence how adolescents develop sexual awareness. Sexual education represented a reliable source of information about sex. Adolescents challenged social and cultural norms by developing sexual agency

## Abbreviations

HIV/AIDS: Human immunodeficiency virus/Acquired immune deficiency syndrome
CHWs: Community health workers
CSE: Comprehensive sexuality education
LMICs: Low- and middle-income countries
NGOs: Non-governmental organizations
SRH: Sexual and reproductive health
PRISMA: Preferred reporting items for systematic reviews and meta-analyses guidelines
SRHE: Sexual and reproductive health education
SRHR: Sexual and reproductive health education rights
STIs: Sexually transmitted infections

## References

[1] Yakubu I, Salisu WJ (2018). Determinants of adolescent pregnancy in sub-Saharan Africa: a systematic review. Reprod Health.

[2] Herat J (2018). The revised international technical guidance on sexuality education-a powerful tool at an important crossroads for sexuality education. Reprod Health.

[3] Browes NC (2015). Comprehensive sexuality education, culture and gender: the effect of the cultural setting on a sexuality education programme in Ethiopia. Sex Educ.

[4] Starrs AM (2018). Accelerate progress—sexual and reproductive health and rights for all: report of the Guttmacher-Lancet Commission. Lancet.

[5] Kassa GM (2018). Prevalence and determinants of adolescent pregnancy in Africa: a systematic review and meta-analysis. Reprod Health.

[6] Walker J-A (2012). Early marriage in Africa–trends, harmful effects and interventions. Afr J Reprod Health.

[7] Cappa C (2012). Progress for children: a report card on adolescents. Lancet.

[8] Azevedo WFD (2015). Complications in adolescent pregnancy: systematic review of the literature. Einstein (São Paulo).

[9] McClure C, Chandler C, Bissell S (2015). Responses to HIV in sexually exploited children or adolescents who sell sex. Lancet.

[10] Zulu JM (2019). Why teach sexuality education in school? Teacher discretion in implementing comprehensive sexuality education in rural Zambia. Int J Equity Health.

[11] Haberland NA (2015). The case for addressing gender and power in sexuality and HIV education: a comprehensive review of evaluation studies. Int Perspect Sex Reprod Health.

[12] Amaugo LG (2014). The effectiveness of HIV/AIDS school-based sexual health education programmes in Nigeria: a systematic review. Health Educ Res.

[13] Vanwesenbeeck I (2016). Lessons learned from a decade implementing comprehensive sexuality education in resource poor settings: the world starts with me. Sex Educ.

[14] Reiss M (1993). What are the aims of school sex education?. Camb J Educ.

[15] Keogh SC (2018). Challenges to implementing national comprehensive sexuality education curricula in low- and middle-income countries: case studies of Ghana, Kenya, Peru and Guatemala. PLoS ONE.

[16] Chandra-Mouli V, Plesons M, Hadi S, Baig Q, Lang I (2018). Building support for adolescent sexuality and reproductive health education and responding to resistance in conservative contexts: cases from Pakistan. Sex Educ Pakistan.

[17] Manguvo A, Nyanungo M (2018). Indigenous culture, HIV/AIDS and globalization in Southern Africa: towards an integrated sexuality education pedagogy. Handbook of Cultural Security.

[18] Herat J (2018). The revised international technical guidance on sexuality education—a powerful tool at an important crossroads for sexuality education. Reprod Health.

[19] Thin Zaw PP (2021). Abstinence-only or comprehensive sex education at Myanmar schools: Preferences and knowledge among students, teachers, parents and policy makers. Sex Educ.

[20] Denford S (2015). Review of reviews of school-based interventions to improve sexual health and reduce alcohol misuse. Eur Health Psychol.

[21] Santelli JS (2017). Abstinence-only-until-marriage: an updated review of US policies and programs and their impact. J Adolesc Health.

[22] Ott MA, Santelli JS (2007). Abstinence and abstinence-only education. Curr Opin Obstet Gynecol.

[23] Chavula MP (2021). Experiences of teachers and community health workers implementing sexuality and life skills education in youth clubs in Zambia. Glob Public Health.

[24] Birungi H, et al. Education sector response to early and unintended pregnancy: a review of country experiences in sub-Saharan Africa*.* 2015.

[25] Atun R (2010). Integration of targeted health interventions into health systems: a conceptual framework for analysis. Health Policy Plan.

[26] Moher D (2009). Preferred reporting items for systematic reviews and meta-analyses: the PRISMA statement. PLoS Med.

[27] Law M. et al. Critical review form, qualitative studies. McMaster University. Grimmer K (2004): Incorporating health research methods and biostatistics and evidence-based research. Research Summer School (Ed.) Course Workbook University of South Australia, 1998.

[28] Panchaud C (2018). Towards comprehensive sexuality education: a comparative analysis of the policy environment surrounding school-based sexuality education in Ghana, Peru, Kenya and Guatemala. Sex Educ.

[29] Renju J (2010). Scaling up a school-based sexual and reproductive health intervention in rural Tanzania: a process evaluation describing the implementation realities for the teachers. Health Educ Res.

[30] Kemigisha E (2019). Process evaluation of a comprehensive sexuality education intervention in primary schools in South Western Uganda. Sex Reprod Healthc.

[31] Tabong PT (2018). Acceptability and stakeholders perspectives on feasibility of using trained psychologists and health workers to deliver school-based sexual and reproductive health services to adolescents in urban Accra, Ghana. Reprod Health.

[32] Ram S, Andajani S, Mohammadnezhad M (2020). Parent's perception regarding the delivery of sexual and reproductive health (SRH) education in secondary schools in fiji: a qualitative study. J Environ Public Health.

[33] Gudyanga E, de Lange N, Khau M (2019). Zimbabwean secondary school guidance and counseling teachers teaching sexuality education in the HIV and AIDS education curriculum. Sahara J.

[34] Emambokus WBS, Oogarah-Pratap B (2019). Exploring parents’ and teachers’ perspectives about school-based sexuality education in a multicultural context: a case study in mauritius. Educ Process.

[35] Svanemyr J, Baig Q, Chandra-Mouli V (2015). Scaling up of life skills based education in Pakistan: a case study. Sex Educ.

[36] Kunnuji MO, Robinson RS, Shawar YR, Shiffman J (2017). Variable implementation of sexuality education in three Nigerian states. Stud Family Plan..

[37] de Haas B, Hutter I (2019). Teachers' conflicting cultural schemas of teaching comprehensive school-based sexuality education in Kampala, Uganda. Cult Health Sex.

[38] Chau K (2016). Scaling up sexuality education in Senegal: integrating family life education into the national curriculum. Sex Educ.

[39] Chirwa-Kambole E (2020). Acceptability of youth clubs focusing on comprehensive sexual and reproductive health education in rural Zambian schools: a case of Central Province. BMC Health Serv Res.

[40] Le Mat MLJ (2019). Moulding the teacher: factors shaping teacher enactment of comprehensive sexuality education policy in Ethiopia. Compare.

[41] Ogolla MA, Ondia M (2019). Assessment of the implementation of comprehensive sexuality education in Kenya. Afr J Reprod Health.

[42] Ha TTT, Fisher JR (2011). The provision of sexual and reproductive health education to children in a remote mountainous commune in rural Vietnam: an exploratory study of parents' views. Sex Educ.

[43] Makleff S (2020). Preventing intimate partner violence among young people—a qualitative study examining the role of comprehensive sexuality education. Sex Res Social Policy.

[44] Adekola AP, Mavhandu-Mudzusi AH (2021). Addressing learner-centred barriers to sexuality education in rural areas of south africa: learners' perspectives on promoting sexual health outcomes. Sex Res Social Policy.

[45] Pokharel S, Adhikari A (2021). Adolescent sexuality education in nepal: current perspectives. Creat Educ.

[46] Bylund S (2020). Negotiating social norms, the legacy of vertical health initiatives and contradicting health policies: a qualitative study of health professionals' perceptions and attitudes of providing adolescent sexual and reproductive health care in Arusha and Kilimanjaro region, Tanzania. Glob Health Action.

[47] Melgar JL (2018). Assessment of country policies affecting reproductive health for adolescents in the Philippines. Reprod Health.

[48] Mhlauli MB, Muchado JA (2015). Hearing voices inside schools: issues of sexuality in upper classes in primary schools in Botswana. J Educ Hum Dev.

[49] Zulu JM (2021). Barriers and facilitators for integration of guidelines on operating health shops: a case of family planning services. J Pharm Policy Pract.

[50] Tetui M (2017). Experiences of using a participatory action research approach to strengthen district local capacity in Eastern Uganda. Glob Health Action.

[51] Schneider H (2022). The multiple lenses on the community health system: implications for policy, practice and research. Int J Health Policy Manag.

[52] Muhwezi WW (2015). Perceptions and experiences of adolescents, parents and school administrators regarding adolescent-parent communication on sexual and reproductive health issues in urban and rural Uganda. Reprod Health.

